# EVITA 2.0, an updated framework for understanding evidence-based mental health policy agenda-setting: tested and informed by key informant interviews in a multilevel comparative case study

**DOI:** 10.1186/s12961-020-00651-4

**Published:** 2021-03-10

**Authors:** Nicole Votruba, Jonathan Grant, Graham Thornicroft

**Affiliations:** 1 Centre for Global Mental Health and Centre for Implementation Science, Health Service and Population Research Department, Institute of Psychiatry, Psychology & Neuroscience (IoPPN), King’s College London, David Goldberg Centre, De Crespigny Park, Denmark Hill, PO Box 28, London, SE5 8AF UK; 2 Policy Institute At King’s, Virginia Woolf Building, The Strand, King’s College London, London, UK; 3 Centre for Global Mental Health and Centre for Implementation Science, Health Service and Population Research Department, Institute of Psychiatry, Psychology & Neuroscience (IoPPN), King’s College London, London, UK

**Keywords:** Knowledge translation and exchange, Evidence-informed policy-making, Agenda-setting, Framework, Evidence-based policy-making, Mental health, Low- and middle-income countries, South Africa

## Abstract

**Background:**

Mental health remains a neglected issue on the global health policy agenda, particularly in low- and middle-income countries (LMIC), and the translation of research evidence into policy and practice is slow. The new EVITA framework was developed to improve mental health evidence uptake and policy agenda-setting in LMICs. In addition, behavioural science methods may be able to support knowledge translation to policy.

**Methods:**

Using a mixed-methods study design, we applied and tested the newly developed EVITA 1.1 framework against three case studies related to South Africa at the district, national and international levels. In-depth interviews with 26 experts were conducted between August and November 2019, transcribed, coded and analysed in NVivo, using iterative categorization. The data were analysed against both the EVITA framework and the MINDSPACE framework for behavioural insights.

**Results:**

In our case study comparison, we found that (1) research translation to the policy agenda occurs in a complex, fluid system which includes multiple “research clouds”, “policy spheres” and other networks; (2) mental health research policy agenda-setting is based on key individuals and intermediaries and their interrelationships; and (3) key challenges and strategies for successful research to policy agenda impact are known, but are frequently not strategically implemented, such as including all stakeholders to overcome the policy implementation gap. Our data also suggest that behavioural science methods can be strategically applied to support knowledge translation to policy agenda-setting.

**Conclusion:**

We found that the EVITA framework is useful for understanding and improving mental health research policy interrelationships to support evidence uptake to the policy agenda, and that behavioural science methods are effective support mechanisms. The revised EVITA 2.0 framework therefore includes behavioural insights, for improved mental health policy agenda-setting in LMICs. More research is needed to understand whether EVITA can be applied to other LMICs and to high-income contexts.

## Background

Mental health has been defined as a “wicked” policy problem, meaning it is often seen as resistant to policy changes and solutions are contestable [1]. In low- and middle-income countries (LMIC) where most people with mental illnesses live, up to 85% of people with such conditions do not receive treatment, yet mental health is often not a policy priority, which perpetuates this wicked problem [2–4]. A number of efforts have been made to increase the relative priority given to mental health in recent years [5–7].

*Knowledge translation* is increasingly making important contributions to accelerate the implementation of mental health evidence into research, policy and practice [8]. Knowledge translation includes a range of concepts and efforts aiming to improve evidence-based policy-making and implementation in practice [9]. Despite increasing research the translation of evidence remains challenging [10–12]. A recent systematic review found that few relevant and practical frameworks exist that address the specific complexities and cross-links of mental health, and support evidence translation to policy in LMICs [13, 14]. The diffuse and constantly changing, amorphous nature and hard-to-define boundaries of research, also described as “*research clouds*”, may contribute to this challenge [15].

A novel method has been introduced to improve evidence translation, evidence-based mental health policy-making and policy outcomes in the field of mental health: The EVITA (EVIdence To policy Agenda-setting) framework [16]. EVITA is unique because it was specifically developed to address the mental health research–policy process in LMICs and to target the *policy agenda-setting* stage. EVITA was validated for mental health-specific factors in LMICs and mental health policy issue priority-setting [16]. In policy studies, agenda-setting was found to profoundly affect policy decisions [17]. The definition in global health is the process through which global health issues gain attention from actors who control/influence the allocation of resources [18]. The EVITA framework specifically defines agenda-setting as “the policy pre-decision process when a problem is identified, defined and prioritized, gains and maintains attention of policy-makers, and eventually becomes a policy priority” [16]. Due to its distinct design and mechanisms targeting the agenda-setting stage, EVITA differs from other frameworks that are focused on implementation into practice [16, 19, 20], and is uniquely suited to analyse mental health research evidence interrelationships with the policy-agenda in LMICs.

When it comes to research impact, research is inevitably intertwined with politics and persuasion [21]. Recently calls have been made that in order to maximize the use of research evidence in policy, researchers should engage more strategically and "recognise the tendency of policymakers to base judgements on their beliefs, and shortcuts based on their emotions and familiarity with information” [22]. In the past decade, behavioural psychology, behavioural science, behavioural economics methods or “*behavioural insights*” have been applied to public policy-making to increase the impact and improve population health outcomes [23]. A number of useful frameworks and tools have been developed to guide behavioural interventions, such as the EAST framework [24] or the COM-B framework and the behaviour change wheel [25]. The MINDSPACE framework aids the application of behavioural science to the policy-making process along nine behavioural influences [26]. Recently, the use of mental shortcuts, such as narratives and storytelling, has been explored to impact policy-making; the results have found that more rigorous research supporting the impact on health policy-making is needed [27]. To our knowledge, no study has yet investigated the use of behavioural science methods in mental health policy agenda-setting.

In response to this gap, the overall aim of this study is to apply and empirically test the EVITA framework against three case studies related to South Africa at district, national and international levels. In a comparative case study, we will test and analyse EVITA’s components and mechanisms, to understand differences in the projects in their interrelationships for mental health agenda-setting in South Africa, and which conditions and behaviours are supporting research evidence and policy interrelationships. The objectives are:

1. To apply, test and analyse the research evidence to policy agenda-setting process against the validated EVITA framework, using the PRIME, Emerald and FundaMentalSDG projects as case studies.
2. To identify which mechanisms, qualities and behaviours were helpful in the interrelationships of research evidence and policy agenda-setting.
3. To identify whether any behavioural incentives were applied, and whether they were effective for improving evidence to policy agenda-setting.

## Methods

This study applies the EVITA framework to three case studies related to South Africa, on the national, district and international levels. We conducted in-depth interviews (IDIs), triangulated the data with a document analysis, and analysed and compared the findings.

### Data collection and analysis

We performed a stakeholder analysis and purposive sampling to identify key stakeholders involved in the three projects. We aimed to gain a representative sample for each study, which included researchers, policy-makers and people working in nongovernmental organizations (NGOs) and international organizations, and to reach saturation. Based on the EVITA 1.1 framework [16], we designed topic guides for each group and piloted the interviews in August 2019. Semi-structured IDIs with 21 experts were conducted by NV in August–November 2019, via Skype, WhatsApp call or in person, for one hour on average. Through snowball sampling we identified and conducted five additional interviews with experts working on LMIC mental health research and policy interrelationships, which were not included in the case studies, but were used to triangulate and cross-validate our case study findings. In total, between July and November 2019, we invited 43 individuals to participate and conducted 26 interviews (62% participation rate). Ethical clearance was obtained from King's College London Research Ethics Committee. Written informed consent was obtained from all interviewees.

All interviews were digitally recorded, transcribed, anonymized and double-coded by NV and a research assistant, using NVivo 12 Pro qualitative data analysis software. Based on the EVITA framework, we developed an a priori coding framework, as recommended for designs that are testing theory against empirical data [28]. In addition to our deductive coding approach, we added further codes as and when they emerged from inductive coding. We analysed our data using iterative categorization (IC) [29].

A narrative review of behavioural science methods was performed. Based on this, the MINDSPACE framework was selected and applied to identify behavioural methods used in the case studies [30].

Finally, we triangulated our findings with document analysis, which included published research papers, publicly accessible records and training material (such as press releases, policy briefs and YouTube videos). These other sources are available from the lead author on request.

### Case study analysis

We performed an explanatory comparative case study to analyse and contrast how—that is, through which stakeholders and processes—research evidence was translated on the policy agenda, and to uncover which contextual conditions and behaviours were relevant to research evidence and policy interrelationships.

The three projects were selected because all three were active around the same time, were working on mental health systems research implementation/advocacy, and had a strong focus on research evidence and policy interrelationships. All three were active in the same sociopolitical context (South Africa), on three different levels: PRIME worked on the district level (micro), Emerald on the national level (meso) and FundaMentalSDG on the global level (macro). All three projects had the wider aim to contribute to improving care and reducing the treatment gap for mental health conditions [31–33]. All projects were multiple-country initiatives; two of them were research collaborations (Emerald and PRIME), and FundaMentalSDG was a global programme to set the United Nations (UN) agenda. An overview of the case studies is presented in Table 1.

**Table 1:** Overview of case studies

Project	Level	Implementation site	Funder	Active	Key publications	Aim	Focus
PRIME—Programme for improving mental healthcare	District	Dr Kenneth Kaunda (DKK) district, North West Province	UK Department for International Development (DfID)	2011–2019	(30, 32)	To support the integration and scaling up of mental health care services into primary care	On comorbidity in chronic disorders, and focused on integrating care for depression, alcohols use disorders and schizophrenia into chronic care
Emerald—Emerging mental health systems in low- and middle-income countries	National	Dr Kenneth Kaunda (DKK) district, North West Province	European Commission FP7	2012–2017	(29, 33)	To scale up services and enhance mental health outcomes by improving mental health systems performance across six LMICs	On building capacity and generating evidence to strengthen health systems and mental health care on the national level
FundaMentalSDG initiative	International	UN Sustainable Development Goals (SDGs)/global	Unfunded	2014–2016	(31, 34)	To include and strengthen mental health in the UN SDG goals, targets and indicators, i.e. to support setting the UN SDG agenda	Using a wide global network of mental health experts and organizations in research policy and practice to impact the policy agenda based on research evidence, targeting UN missions and nations’ ministries

## Results

This section presents an overview of our sample of in-depth interviews (see Table 2), the changes that we made, the revised EVITA 2.0 framework and a new process diagram. Lastly, we present our key findings.

**Table 2:** Characteristics of in-depth interview sample (n = 21)

Category of key informant	n =	PRIME (district)	Emerald (national)	FundaMentalSDG (international)
Research cloud	15	10	7	1
Policy sphere	3	2	0	2
Civil society organization/NGO	1	1	0	1
External stakeholder	3	1	0	1
Total	21	14	7	5

Some interviewees were involved in, and interviewed for, more than one project

### Overview of our interview sample

#### Changes following the interviews

Before presenting the key findings, it is important to acknowledge the changes we made to the EVITA framework as a result of the key informant interviews. We analysed the interviews in the context of the case studies we were exploring, based on the EVITA 1.1 framework. Overall, the EVITA framework was helpful in analysing the three case studies. In doing so, a small number of issues arose that allowed us to strengthen the framework, and led to the following minor changes in the EVITA framework:

1. We added *key individuals*
2. *Intermediaries* tend to occur in all stakeholder groups and dynamically move between them
3. We renamed *evidence generators* to *research cloud*
4. We renamed and moved *external influences* into *external context*
5. *Catalysts* are part of *external context*
6. We renamed *communication, relationship and partnership building* to *engagement & relationships*
7. We renamed *framing* to *framing & alignment*
8. *We renamed the political context* to *policy sphere*
9. We added *behavioural incentives* as a mechanism
10. We organized all components and mechanisms into four categories (see Additional file 1)

#### The new EVITA 2.0 framework

**Fig. 1:**
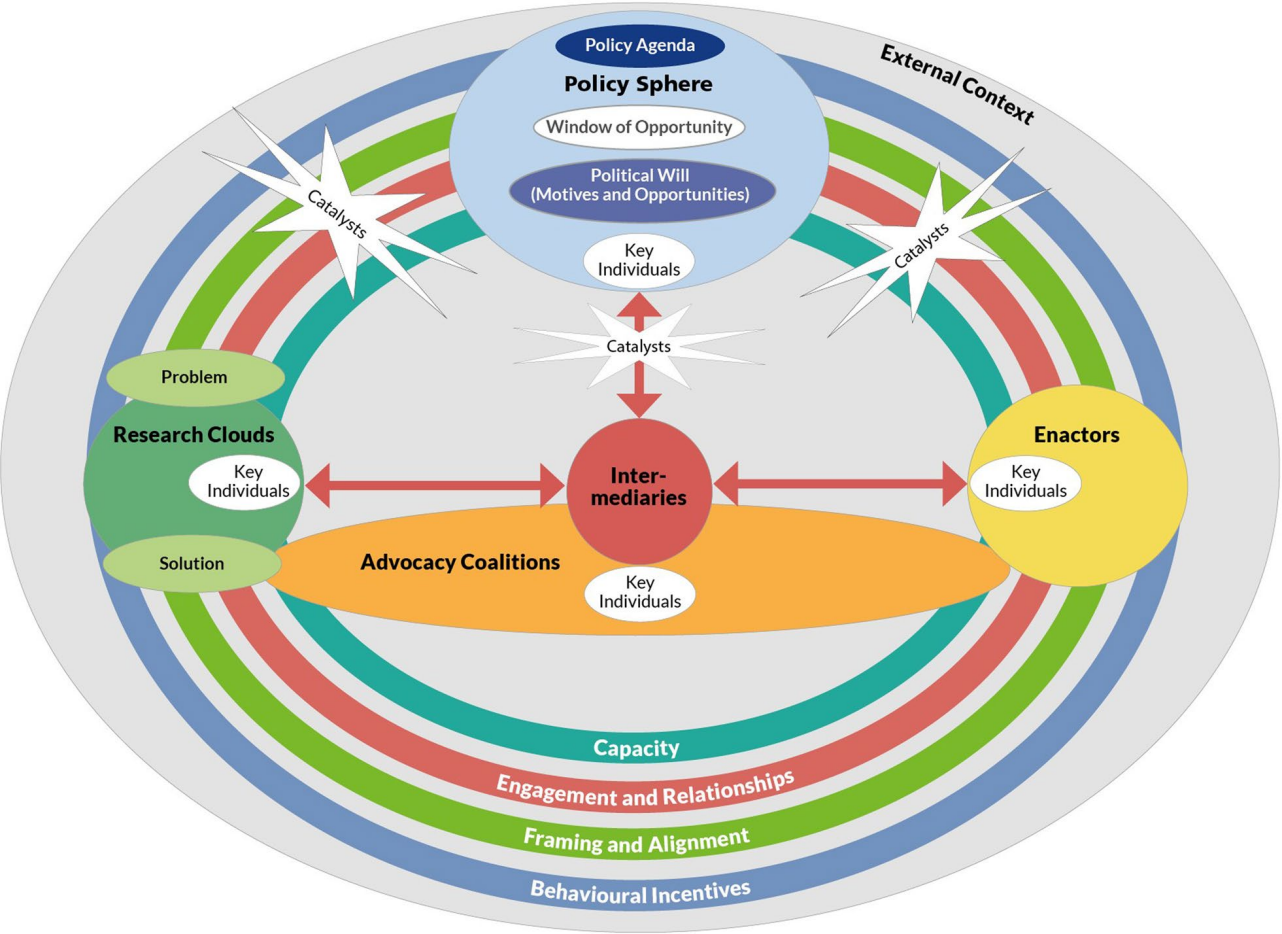
The new EVITA 2.0 framework

The new EVITA 2.0 framework presented in Fig. 1 includes the changes based on our findings.

**Fig. 2:**
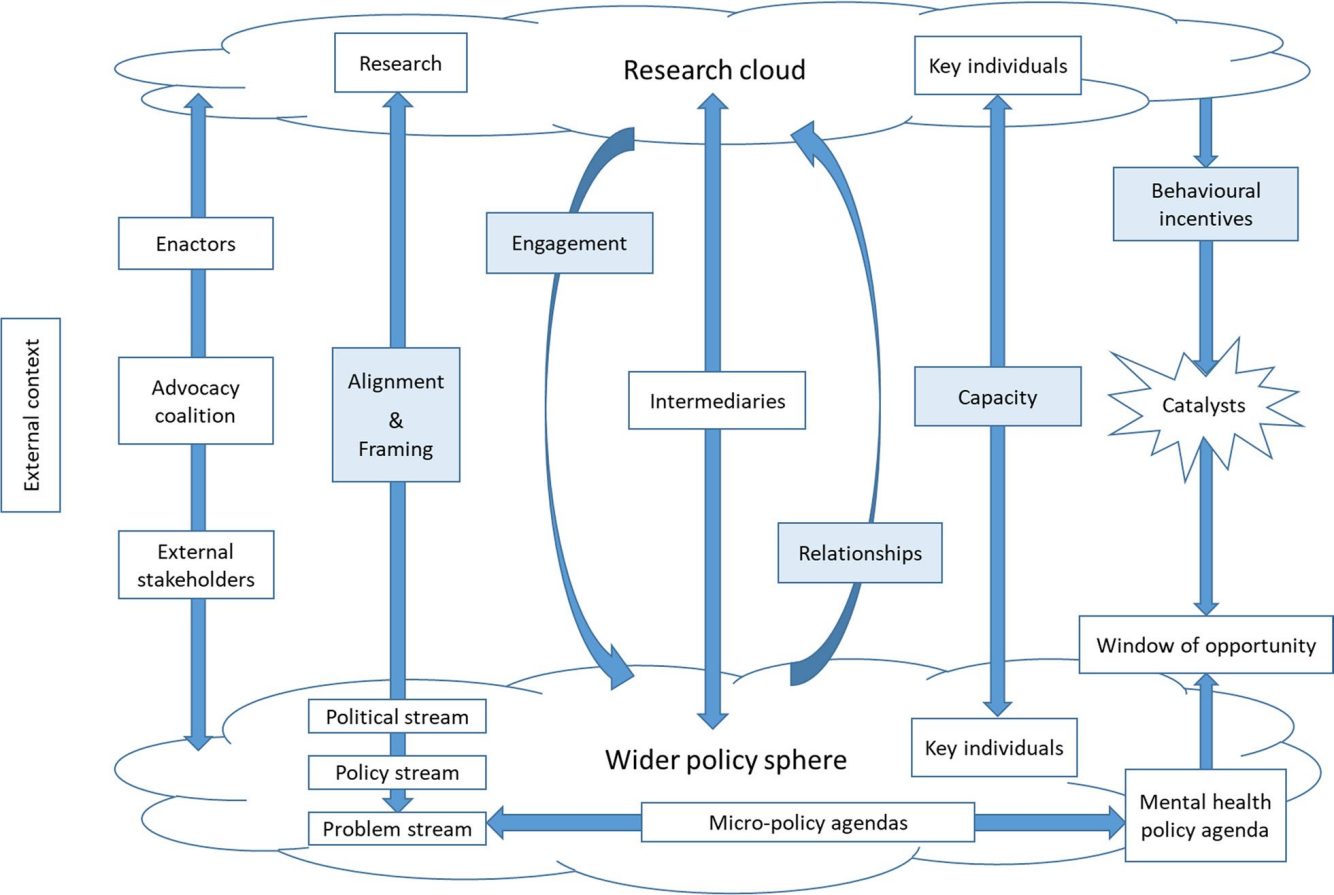
EVITA process diagram

We added a process diagram to illustrate the fluid dynamics of the research-policy evidence exchange in EVITA (see Fig. 2).

### Key findings

From the qualitative analyses of the case studies, we derived three main themes with three key findings for each theme, which are summarized here. Details and definitions for all elements of the EVITA framework (indicated in italics) can be found in Additional file 1.

## Research translation to the policy agenda occurs in a complex, fluid system which includes multiple “research clouds”, policy spheres and other networks

### Research-policy translation in mental health occurs in a complex system, better defined through networks than linear pathways. It is characterized by fluidity across the system and all elements

Research evidence to policy agenda-setting seems to take place in fluid, dynamic and multilevel processes, spanning various networks of the research-policy-practice system. Research impacting on the policy agenda seems to stem from diffuse evidence clusters or “*research clouds*” with fluid boundaries rather than single research projects [15] which are centred around key individuals. For instance, the Emerald and PRIME programmes were developed within “clouds” of preceding, parallel and follow-on research projects, and key stakeholders in South Africa and globally were/are involved in and across those projects, ranging beyond the scope of mental health, into physical health and even beyond health.

FundaMentalSDG accessed findings from those research clouds, but, because of its global aim, spanning a greater number of them. Stakeholders are shifting through those clouds/networks and need to engage across all levels: “*That can only happen through working with your people from national through to provincial level through to district level. […] You can’t just work at one level; you should actually ideally be working at all levels*” (researcher, ID18). Changes in people, budget and events occur frequently and can influence the translation substantially. Several interviewees described research translation as a serendipitous event, uncontrollable and unpredictable, resulting from a summary of influences. However, we found that with “*serendipity*”, ultimately they seem to be referring to those unstable, changing networks, relationships, and events: “*One of the things you can't discount is serendipity. There are actors and players in this whole scenario who may be, at a particular point in time, holding key positions*” (researcher, ID9). Advocacy coalitions seem to be capable of helping strengthen and stabilize the system, and ideally are directly linked to research clouds through key individuals.

### Policy agenda-setting is a complex, nontransparent process. Understanding the system, mechanisms, stakeholders and context is vital, yet time-intensive

Mental health policy agenda-setting and decision-making are cyclical, back and forth processes, embedded in networks of relationships across all levels, within and outside policy. The structures, processes, and stakeholders are nontransparent, and hard to understand and penetrate for researchers and others engaging in research translation and policy agenda-setting. Interviewees repeatedly stated that for UN outsiders, the UN Sustainable Development Goals (SDG) agenda-setting and policy development was highly nontransparent, and understanding how to gain access and influence was difficult and tedious. Even insiders admitted that “*the process for SDG discussions was not completely transparent and also not very easy to negotiate”* (policy-maker, ID20). Access was only successful through knowledge from inside-experts (*intermediaries*), and *“it took a lot of excavation to find out, just even specifically for Goal 3 or the health-related goals, and then the targets and the indicators (…), what had gone before, who had been involved, going back how long, and how we could understand which views, voices and pressures had been incorporated along the way”* (researcher, ID4). Data from PRIME and Emerald confirmed these problems on the district and national levels.

*Capacity* for translating evidence, and for recognizing windows of opportunity is vital: understanding the political, policy and implementation context; external influences such as the health care context; key people, events, time frames and (competing) priorities; and the ability to frame the evidence accordingly. Supporting policy-makers beyond the project realm and mental health appeared as a helpful mechanism: “*We’re not just a research organization, my unit, but we actually provide services to the department. We don’t just develop programmes, we actually help the department in the programmes we develop, and eventually we gain some level of interdependence from that*” (researcher, ID24). Capacity also includes a good understanding of key individuals and policy-makers, and their priorities and needs. “*To have EQ in terms of your interactions […and] a high level of empathy*” (researcher, ID24) emerged as supportive capacity for working with policy and improving research uptake on the policy agenda.

### Agenda-setting for mental health occurs on multiple micro-policy agendas. Research translates most effectively when it is multisectoral and aligned to other (health) policy priorities

Agenda-setting for mental health occurs on the mental health policy agenda, but simultaneously also on other, often competing, policy agendas. For instance, in the SDG process, overall development issues competed for the limited targets which aimed to “*leave no one behind*”, health conditions within the health goal, and even mental health conditions amongst one another. *“There were other departments that were looking for their own targets and goals and also eventually indicators. So, this was a very complex process*” (policy-maker, ID20).

The accounts of participants revealed that research translation is most effective when the evidence is aligned with, and framed to, multiple micro-agendas beyond mental health. PRIME’s research and implementation was rather carefully mapped out and aligned with current policy issues and needs. *“We realized that simply being in mental health was an awful waste of time. One had to be in both contexts where HIV, TB, chronic illnesses, other chronic illnesses, all of those were part of the conversation and that all of those people were in the same room. If you wanted mental health to be integrated, you would need to talk to that whole group. (…) We bypassed the policy-making connection that research into policy and then discussing with policy makers about how they could support and initiate some sort of implementation”* (researcher, ID9). Emerald also aligned the programme to support policy-makers’ needs. FundaMentalSDG framed policy solutions to the WHO Mental Health Action Plan 2013–2020 [37] and wider societal cross-links.

Participants emphasized that engagement across these agendas is a substantial effort, requiring constant adaptation and negotiation. “*Political will*” for taking action emerges within this dynamic process, but needs to manifest and sustain on a critical number of those micro-policy agendas. *“What you need is a good nucleus of mental health people within health to be able to convince the other departments and ministries to take more interest”* (policy-maker, ID20).

## Mental health research policy agenda-setting is based on relationships, key individuals and intermediaries

### *Relationships are the central and most critical mechanism* for translation and uptake of mental health research on the policy agenda

Our interviews revealed that *relationships* are the most critical mechanism for research to policy agenda-setting. Research translates through engagement of stakeholders within long-standing, well-fostered relationships, and is critically influenced by their quality. *“If it's a person that you've got good relationships with, you do take them very seriously*” (policy-maker, ID11).

These relationships appear to be largely independent of research programmes, and rather linked to key stakeholders, who continue to build and maintain those relationships before any particular projects starts, and beyond the project endings. Emerald and PRIME research was substantially embedded in pre-existing relationships, rather than relationships developing around the research: *“Obviously, there were direct approaches to policy makers within South Africa. They were done as part of ongoing relationships that had been developed by the South Africa team. They’d been working with those policy makers on different things over extended periods of time. The exchange of information about PRIME and Emerald was rather embedded within those relationships”* (researcher, ID6).

PRIME interviewees emphasized how they very carefully cultivated, and invested in, their relationships with policy-makers, and other stakeholders: *“I invest very deeply in my relationships with the policy-makers. I have them on speed dial. So, there’s a lot of contact”* (researcher, ID24). Interviewees stressed the critical relevance of relationships, to make or break processes in policy and practice. Continuous, long-term engagement enables access to critical events (such as committees, task groups) and opportunities for research-policy agenda-setting. *“It’s building long-term relationships and saying that 20% of my time, or whatever it is, is going to have to be on those relationships”* (researcher, ID5). Even training “*was much more determined by people just building lots and lots of relationships with policy-makers*” (researcher, ID8). In global networks, such as for FundaMentalSDG, it appeared more difficult to establish relationships and engage at short notice.

### *Key individuals* are vital in initiating and driving research-policy translation, and function as major agents for change

*Key individuals* are key connection points within research clouds, policy and practice and a vital element for research translation. They are leading the research, have high *capacity*, and have a wide, high-quality network of *relationships*, through which they enhance collaboration and research uptake. *“In South Africa, [the senior researcher] is known by everybody involved in mental health. He’s built relationships over 10 years. When he comes out with a finding, he can talk to them, and then they go, ‘That’s a good idea.’ They, mostly, are not going to read the paper. Mostly, they don’t know how that will necessarily be translated. [He] can help them translate it. It’s relationship, relationship, relationship”* (researcher, ID5).

Key individuals are moving as *intermediaries* through networks, building collaborations and establishing new relationships. Through longstanding, active engagement and fostering of policy networks, they acquired superior access to policy-makers, the policy agenda and potential windows of opportunity. *“Sometimes decisions are hinged around a couple of individual people. There have been some unexplainable situations where people have completely reverted from being quite obstructive to being completely open and supportive and facilitatory*” (researcher, ID14). Key individuals appear to be more relevant for the research-policy agenda translation process, than the projects they are leading. *“Policy-makers don’t remember projects but they remember people”* (researcher, ID12).

In all three projects research and implementation were led by extremely dedicated, committed individuals, driven by a high personal commitment to research impact, and the desire to influence the policy agenda and health outcomes. Interviewees described research translation as “*incremental pieces of work—persistence*” (researcher, ID6).

In Emerald and PRIME, key individuals were substantially influencing capacity-building of junior colleagues and policy-makers, primarily by working with them, rather than formal training. FundaMentalSDG was a “learning by doing” capacity undertaking, based on the experience and knowledge of key individuals.

### *Intermediaries* are vital in allowing people to navigate the system. They are found on all levels of the system

*Intermediaries* are the link to access critical networks, key individuals, and events for enabling research policy agenda-setting [16]. Intermediaries are moving fluidly across all levels of the system, and can hold, and switch between, other roles, such as *key individual* and gatekeeper in policy: “*I didn't think I was a policy-maker. (…) As much as I would have wanted to do, I would have had to get through the minister and (…) the National Health Council, which was, basically, the provincial ministers and the minister, who would all then have to make a decision that 'that's where we're going to go'*” (policy-maker, ID11).

Intermediaries have relationships with key people, and are critical for enabling access to the policy agenda. They have capacity in understanding the system and its pathways, and can frame and translate evidence between the different connection points. They build capacity in other stakeholders and key individuals, and act as enabler or “*directed catalyst*” for research policy agenda translation. “*Having [Dr L], who knows people in the Department of Health really well, and how they think and function, getting guidance from her on how to present stuff and how to work with people (…) is useful*” (researcher, ID18). They can be engaging with stakeholders as champions: “*[They] had some very key people (…) not within the project, but they had them on the advisory group, who were very supportive of PRIME and really trying to just champion the agenda of PRIME over a long period of time*” (researcher, ID8). Also, FundaMentalSDG worked strategically with intermediaries from the beginning, for “*accelerating the process of making a connection*” (researcher, ID4). Changes in intermediaries affected relationships and research translation: “*She did retire a few years on and (…) we were not able to engage with anybody else after that sort of at her particular level*” (researcher, ID16).

## Key challenges and strategies for successful research to policy agenda impact are known, but frequently not strategically implemented

### Key individuals know mechanisms for effective research translation. Yet, these are often only reflected in hindsight, rather than implemented following a clear *engagement strategy*

We found that key individuals had good theoretical *capacity* (knowledge, experience and ability) for effective research translation and policy agenda-setting. However, upon reflection, most interviewees expressed that they were not always implementing their capacity most effectively, and often reflected on what should have happened for the benefit of hindsight. We found that all three projects were familiar with best-practice strategies for evidence translation and uptake on the policy agenda. PRIME and Emerald both had a research uptake strategy, however, for instance, several interviewees found policy briefs to be ineffective: “*I think policy briefs do not work. They're great to put your coffee on*” (researcher, ID18). Yet, throughout all projects, a main mechanism was “*providing evidence through policy briefs to the policy-makers”* (researcher, ID9).

It appeared that engagement strategies were shaped by funders’ requirements, which at times seemed to deflect from substantial research-policy engagement, reducing the impact on the completion of a deliverable. Engagement was used mostly as an implicit part of the project work rather than as a strategy in its own right. In PRIME, training was mostly performed implicitly, within existing research-policy relationships of key individuals, yet interviewees found that more capacity-building would have been helpful. Emerald interviewees reported that training should have been better adapted to the country context so the impact could have been increased. Relationships seemed to be more relevant/catalytic in translating evidence into practice. *“From the lessons that we learned in Doctor Kenneth Kaunda through the PRIME project, we realized that one of the biggest things to do was to really strengthen our engagement so that we get proper buy-in. Not be so heavily involved, ourselves, on the ground, but to help capacitate the people on the ground”* (researcher, ID2).

For all three projects, a lack of resources was identified as a problem in delivering a coherent and comprehensive engagement strategy.

### The *policy implementation gap* is one of the biggest challenges, but other vital stakeholders are frequently being left out of the research design and policy translation process

Many interviewees identified translating research evidence and national policies down to provincial and district level as a key problem in South Africa: *“The barrier is how do those central federal level policies get translated down to the provincial governments, who may not share the vision and may have many other competing health problems that may be more localized and affect their population more in the provinces”* (researcher, ID6). The implementation context had a substantial impact on policy agenda-setting and implementation, for example, initiated through active engagement in practice (e.g. creating demand for services), via external stakeholders (e.g. evidence-based WHO guidance documents) or through external influences (e.g. corruption, implementation readiness). Research funders were found to have a considerable influence on the programme structure and implementation. However, rarely were external stakeholders significantly involved in the research design, implementation, translation or policy agenda-setting process.

Training aimed to bridge the policy–implementation gap and engage enactors, but it seemed that engagement was more effective *“we had meetings and we had almost monthly (…) engagement with them, (…) just to try and keep our presence sort of stable in the district and for people to get accustomed to this concept of mental health and to understand what we're trying to do*” (researcher, ID16). The uptake of research in practice was also found to be a major gap in FundaMentalSDG. During the agenda-setting and policy formulation process, the SDG implementation was not sufficiently clarified, so now the SDGs “*fall short at the level of implementation or practice*” (researcher, ID9). This may substantially limit the impact of the mental health targets, also within the all-inclusive SDG agenda, which lets countries set priorities and likely rather choose easily achievable targets, than *“mental health [, where…] the evidence and data are still inadequate”* (policy-maker, ID20).

### *Behavioural methods* can be strategically applied to strengthen research to policy agenda-setting

In addition, we found that all projects applied behavioural economics methods to increase the uptake of research on the policy agenda. We identified use of all behavioural influences described in the MINDSPACE framework (see also Additional file 2).

The *messenger* was used unanimously, for instance through involving respected key people, or intermediaries linking research to policy-makers: *“I get her to present stuff sometimes, because they trust her more than they trust me”* (researcher, ID18). PRIME and FundaMentalSDG stressed the influence of VIPs, and the impact that a lack of involving these would have. *Incentives* were used in Emerald and PRIME, for example by taking policy-makers’ and other stakeholders’ needs into consideration, or communicating promising results and “*anything really that we felt that would give the Department of Health some positive information about the progress of the research”* (researcher, ID16). In FundaMentalSDG we found that *norms* were relevant for agenda-setting: “*The [UN] would then (…) receive that proposal, and reflect on how many people were signing up to this proposal and who they were. If this came from 150 nations, then they’d take it quite seriously. If it came from two or three small countries, they might not*” (researcher, ID4). FundaMentalSDG also applied *defaults*, by linking the suggested indicators to the earlier agreed WHO Mental Health Action Plan, but without clear positive outcome.

In PRIME, *salience* was used as key mechanism to trigger the Department of Health’s uptake of research on agenda. *Priming* (repeated engagement/perseverance) was applied strongly in Emerald and PRIME, but less in FundaMentalSDG. Also, *affect* was found in Emerald and FundaMentalSDG, such as key people having a personal link to mental conditions. Although both Emerald and PRIME had involved policy-makers from the start, *commitments* were only found in PRIME, possibly because policy-makers were included as collaborators of PRIME from the bid development stage. *Ego* was effectively used in Emerald and PRIME, for instance by giving policy-makers credit for research-policy successes and “*providing opportunities for policy-makers to also be profiled”* (researcher, ID24).

## Other findings

### Engagement with external stakeholders may be vital

The interviews suggest that beyond policy-makers and enactors, engagement with external stakeholders may be vital to accelerate research translation efforts. PRIME and FundaMentalSDG engaged with the media, while service users helped to shift the public image through the media: “*We built quite a good relationship with the media and then when they printed something they would always check with us is it accurately that they're reporting”* (service user advocate, ID13). In addition, it was found that alignment of research proposals with influential external stakeholders, such as the disability community, would have been helpful. Advocacy coalitions could support the process.

### Stigma may be a key barrier

We found indication across all three projects that stigma may be a key barrier for policy-makers and enactors to taking evidence into account and taking action. In South Africa, stigma seems to remain particularly in rural areas, but it was also perceived in past years that there “*has been a shift in orientation to mental health, and an openness*” (researcher, ID14). Yet, global data suggest that stigma may be disguising discrimination as “misconception or unawareness” in policy-makers, and recent studies support this [38].

## Discussion

This study set out to understand research and policy interrelationships for mental health agenda-setting, by applying the EVITA framework to three case studies on the national, district and international levels in South Africa. The key findings were that research translation to the policy agenda occurs in a complex, fluid system of research clouds, policy and other networks; mental health research policy agenda-setting is based on relationships and agents; and researchers usually seem to be aware of key challenges and effective strategies, but rarely implement these strategically. We aimed to answer three main questions: (1) applying the EVITA framework against three case studies, how did research evidence interrelate with policy agenda-setting; (2) which mechanisms and qualities/criteria were helpful; (3) which behavioural methods were used and did they improve the research-policy agenda process?

### How research and policy agenda interrelate

Across all three case studies *relationships* were central to the research-policy agenda-setting process, which is supported by reviews [39]. *Key individuals* are drivers of the knowledge translation process, and supported by *intermediaries* as gate keepers, match-makers and facilitators. Interactions and roles occur in a fluid environment of *research clouds* and *policy spheres*, and our findings suggest that people and relationships have a greater mean lifetime and impact than research projects themselves. The impact of key individuals working across boundaries and, due to different atmospheric conditions, constantly changing *research clouds* was introduced earlier [15].

Overall, we found good coherence across the three case studies. We noted differences where the projects are located within the EVITA framework. Both national (Emerald) and district (PRIME) projects are located within the *research clouds*, whereas the FundaMentalSDG initiative is an *advocacy coalition* accessing and translating the evidence. This strengthens both validity and applicability of EVITA.

Due to the fluid system, *engagement* was difficult to delineate between national, district and international levels. Effective research-policy agenda-setting seems to be required *continuously* across all levels/system, regardless of the implementation level of the project. Researchers should involve policy-makers, enactors and external stakeholders from planning though to implementation phase, in a co-creation process, instead of selective consultations [18, 40]. This can improve health research processes and outcomes and the function of health systems, although challenges for researchers remain [41–43]. *Engagement* and *relationship-building* are vital [44], yet rarely used as a conscious/strategic mechanism in research translation [45, 46].

Our findings suggest that in parallel, researchers should engage with stakeholders to set and align their *micro-policy agendas*. Studies on the effectiveness of global health networks support this finding [47]. *Agenda-setting* also needs expand to address the political, public and media agenda alike. This is applied in communication research [48–50] and in the political sciences, to turn public issues into actionable policy priorities [17]. The EVITA framework addresses the three agendas to maximize the impact of agenda-setting efforts for mental health.

We did not find strong use of *advocacy coalitions* in the two research projects; however, data from FundaMentalSDG suggest that advocacy coalitions can be an important stakeholder for unifying, aligning, framing and translating evidence to the policy agenda [33], and could be more strategically used [5].

### Effective mechanisms and qualities

Our results strongly suggest that *relationships* are the single most important key mechanism for research impact on the policy agenda. Across all three studies, researchers built, reinforced and strengthened relationships with policy, which increased engagement across the wider knowledge ecosystem, and evidence uptake. Earlier studies stress the relevance of ongoing relationships [39, 45, 51, 52], in particular integrated knowledge translation [53], and suggest investment in relationships should be part of a research uptake strategy [54, 55].

The second key mechanism is *framing and aligning* research as a solution to current policy problems and political priorities. This includes existing *micro-policy priorities and agendas*, such as other health urgencies, pressing socioeconomic issues, or educational targets. Our data suggest that this was particularly relevant during the competitive SDG agenda-setting process, where policy stakeholders dreaded the large number of targets and indicators, while mental health evidence was considered to be largely insufficient, unreliable and without adequate measurements. Data fidelity is important [39], but not sufficient on its own [56], and it remains unclear how much decisions are eventually based upon the evidence (quality) or rather its fit into context [57].

Thirdly, a set of qualities was confirmed in all three case studies, summarized as *capacity*: the knowledge of, experience in, and ability for research translation and policy agenda-setting; and the understanding of the policy and implementation context and policy-makers’ needs and priorities. A recent systematic review supports our findings [58]. Capacity-building is the enhancing mechanism, implemented in public health in particular through knowledge translation platforms [59, 60], and materials from high-income country (HIC) contexts need local, contextual adaptation to be effective [11, 61].

### Behavioural methods may be effective agenda-setting mechanisms

In addition to these mechanisms and qualities, we found that researchers utilized a number of *behavioural methods* to enhance research-policy impact. Behavioural insights are effective in improving outcomes of public health policy implementation [25, 62, 63]. And evidence may too often be targeted at decision-makers’ “slow thinking”, rather than “quick thinking” [56, 64]. Our data suggest that behavioural incentives, such as using a messenger, setting clear incentives, applying norms, framing issues, priming, salience, and focusing on policy-makers benefits, affect and commitment, may be effective to improve research uptake. These incentives may help trigger interest and action in policy-makers, in particular when catalysts occur. Further investigation is needed to determine which methods are effective in which audiences under which conditions.

### The revised EVITA 2.0 framework

Our findings suggest some changes to the original EVITA 1.1 framework. *Key individuals* were added, in line with (policy-)entrepreneurs in earlier studies [65, 66]. We adapted the concept of *research clouds* [15], and restructured the components into stakeholders, influences (uncontrollable), mechanisms (controllable) and qualities.

It also became clear that where stakeholders are in the framework depends on the context. For instance, while in the national and regional level case study, the UN are external stakeholders, in the global case study they become the centre of the policy sphere.

EVITA 2.0 was specifically developed to support the uptake of research evidence on the policy agenda for mental health. With its specific focus on policy agenda-setting, EVITA could also complement other frameworks on implementation into practice, and vice versa, in particular for neglected health policy issues. While established implementation frameworks such as the integrated Promoting Action on Research Implementation in Health Services (i-PARIHS) or Consolidated Framework for Implementation Research (CFIR) also stress the key role of individuals and facilitators, they are largely focused on effective implementation relying on the strength of evidence and facilitation in the specific context [19, 67, 68]. The EVITA study has added further evidence to understand and improve the complex, fluid nature of research to policy translation, using strategic translation of evidence amongst key stakeholders and intermediaries, as well as framing, alignment and other behavioural incentives. While this evidence specifically on the research-policy process can complement implementation frameworks, this study has also found a need for addressing the implementation gap already at the research to policy agenda-setting stage. A detailed account of the EVITA framework in relation to other frameworks has been described elsewhere [16].

## Limitations and future directions

This study has limitations and research opportunities. Firstly, the application of the EVITA framework is limited to data from three case studies in South Africa and the global UN SDG context. While we draw some lessons from these, we recognize the limitations of our analysis and emphasize the need for other comparative case studies to test their validity beyond the South African context.

Another limitation arises from our predominantly qualitative approach to testing the framework, which does not enable us to analyse the relative importance of each element. Future studies could quantitatively test which components, mechanisms, influences and capacities are most effective.

An additional limitation is that the majority of informants interviewed were researchers (15/21). We tried to mitigate this by inviting a large number of interviewees from research, policy and practice. Yet, as the response rate from policy-makers was extremely low, the study had to rely on the respondents. Additional interviews were conducted with experts working on LMIC mental health research and policy interrelationships from outside the case studies and were used to triangulate and cross-validate our case study findings.

The focus of our analysis was on *research-policy* interrelationships, not on implementation into practice. Collaboration and alignment with *enactors* is important, yet additional mechanisms may be needed. Engagement with *external stakeholders* is essential, in particular the media for framing the public image [69, 70], and social media channels may also have substantial potential for impact [71]. Our study also confirmed the significant influence of funders/donors [72, 73] and international organizations on the evidence-policy agenda-setting process [74, 75]. Yet, this study did not explore how to best engage and collaborate with them; further research could build on other evidence-based strategies [41]. While our findings are also largely consistent with empirical studies from implementation science, learnings can be drawn from this field, in particular on effective strategies for implementation [76].

We had contradictory findings in regard to stigma. Some data suggested a general shift towards more openness regarding mental health in South Africa, but stigma also seemed to persist, disguised and legitimized behind ignorance. Numerous stigma-reduction interventions have been carried out, yet more evaluation of effectiveness is needed [77, 78].

Further, our interviewees stressed that any translation from one context to the next requires adaptation. Our findings suggest that EVITA may be also applicable to other LMICs, high-income countries, and other low-priority health issues; however, the application and validity of EVITA need to be tested and potentially adapted.

It is also relevant to note that two authors of this study were substantially involved in the projects used as case studies. This was a strength in understanding and conceptualizing the data, and acquiring the bigger picture, but we acknowledge a potential for confirmation bias in relation to our initial hypothesis. We sought to mitigate this by taking a rigid methodological approach, efforts to analyse and interpret our qualitative findings as objectively as possible, for example through the involvement of the co-authors, double-coding, cross-validation with key experts and triangulation with other sources.

## Conclusions

The aim of this study was to apply and empirically test the EVITA framework against evidence from three case studies in South Africa on the district, national and international level. We found that the EVITA framework was helpful to analyse and understand mental health research and policy interrelationships and agenda-setting in South Africa. EVITA helped identify key stakeholders and their engagement as vital, as well as methods, qualities and behaviours for effective interrelationships, such as relationships, alignment, framing and capacity. In addition, our study suggests that adding behavioural methods to a strategic research engagement strategy can help improve research-policy agenda-setting. The new EVITA 2.0 framework includes these elements, and may therefore also be helpful to strategically support evidence uptake into policy and practice.

More research is needed to test EVITA 2.0 in a prospective study, and to understand whether EVITA can be applied to, and effective in, other LMIC contexts, or beyond. A future study could measure and compare effectiveness of individual mechanisms on policy agenda-setting and implementation of evidence. In particular, the use and impact of behavioural mechanisms in research engagement could be investigated further.

### Implications for research, funders and policy

From our findings we draw key implications for research, funders and policy, which can support more effective mental health research translation into policy and implementation into practice: (1) researchers, enactors, external stakeholders and policy-makers should engage and co-create evidence from design to implementation stage; new mechanisms/platforms may be necessary to enable this. (2) Key individuals and intermediaries need support for engagement activities, in terms of both allocation of resources and capacity-building; this should be included in the research design and in researchers’ job descriptions towards their institutions. (3) Mutual understanding of different stakeholders should be fostered, and exchange experiences should be encouraged and rewarded; a more flexible research-policy-practice interface should include, strengthen and innovate intermediary positions such as clinical research fellowships, research-fellowships in policy or visiting fellowships for policy-makers.

## Copyright/license for publication

The Corresponding Author has the right to grant on behalf of all authors and does grant on behalf of all authors, a worldwide licence to the Publishers and its licensees in perpetuity, in all forms, formats and media (whether known now or created in the future), to (i) publish, reproduce, distribute, display and store the Contribution, (ii) translate the Contribution into other languages, create adaptations, reprints, include within collections and create summaries, extracts and/or, abstracts of the Contribution, (iii) create any other derivative work(s) based on the Contribution, (iv) to exploit all subsidiary rights in the Contribution, (v) the inclusion of electronic links from the Contribution to third party material where-ever it may be located; and, (vi) licence any third party to do any or all of the above. According to UK research councils’ Common Principles on Data Policy, all data supporting this study will be openly available at http://doi.org/doi:10.18742/RDM01-123.

## Supplementary information

**Additional file 1.**EVITA 2.0 categories.

**Additional file 2.**The MINDSPACE framework.

## Data Availability

The datasets used and/or analysed during the current study are available from the corresponding author on reasonable request.

## Abbreviations

HIC: High-income countries
LMIC: Low- and middle-income countries
UN SDGs: United Nations Sustainable Development Goals
