# Supplementary material for: EVITA 2.0, an updated framework for understanding evidence-based mental health policy agenda-setting: tested and informed by key informant interviews in a multilevel comparative case study
Source: Health Res Policy Syst. 2021 Mar 10;19:35. doi: 10.1186/s12961-020-00651-4 (PMC7948345; doi:10.1186/s12961-020-00651-4)
Supplement: Supplementary file 1 — Additional file 1.EVITA 2.0 categories. [file 12961_2020_651_MOESM1_ESM.docx]

**Additional file 1: EVITA 2.0 categories**

EVITA’s elements (former components and mechanisms) are now grouped into four categories.

This table illustrates the details for a clearer understanding of EVITA 2.0.

| **Category** | **Description** | **Element** | **Key features** |
| --- | --- | --- | --- |
| **Stakeholders** | They are the actors within the research-policy inter-relationships | Key individuals | Policymakers, researchers, and other relevant people leading the efforts for mental health policy agenda-setting; they are often fluidly moving into roles of intermediaries |
|  |  | Enactors | Implementers of the research, and those directly involved in the implementation, such as service users/groups, carer/family organisations, doctors, NGOs, nurses, service providers, other experts |
|  |  | Intermediaries | Individuals or organisations acting as key linkages/ knowledge brokers between research, policy and/or practice; They have high capacity and engagement, they are often fluidly moving between the spheres |
|  |  | External stakeholders | International organisations such as the UN, WHO, Worldbank, EU; the media; donours and funders; religious leaders, faith groups; trade unions |
| **Influences** | Influences which occur and can usually not/ hardly be actively be directed | External influences | socio-economic environment, health care context, cultural and ideological setting and beliefs; global policies, frameworks and priorities |
|  |  | Catalysts | Usually unforeseen events, changes, which trigger/increase the uptake of research on the policy agenda |
| **Capacity** | Capabilities and qualities which enable ideal conditions for research-policy agenda translation | Experience | Acquired practical and theoretical knowledge |
|  |  | Comprehension | Understanding of political- and policymaking context and implementation context; recognising key individuals (policymakers, intermediaries, enactors) and their priorities, motivations and opportunities, and of windows of opportunity |
|  |  | Qualities of the researcher | credible, renowned in the field and policy, trustworthy and reliable |
|  |  | Qualities of the evidence | clear, understandable, accessible, good quality, rigorous, up to date, timely and relevant |
| **Mechanisms** | Mechanisms with which research translation and uptake on the policy agenda can be actively be stimulated | Alignment | Identifying current (health) policy(makers’) priorities and aligning mental health research to these |
|  |  | Advocacy coalitions | Coalitions based on co-production, with a joint unified ask to the policy agenda |
|  |  | Capacity building | Training to increase knowledge, qualities and mutual understanding of context, research, policy and practice |
|  |  | Behavioural incentives | A set of (non-coercive) tools to influence behaviour, which may have the potential to improve research uptake on the policy agenda |
|  |  | Framing | Adaptation of research to the specific context, time, stakeholders, relevance, health and other policy priorities |
|  |  | Engagement & relationships | Engagement includes general engagement, communication, relationship and partnership building, collaborations |
