# Supplementary material for: EVITA 2.0, an updated framework for understanding evidence-based mental health policy agenda-setting: tested and informed by key informant interviews in a multilevel comparative case study
Source: Health Res Policy Syst. 2021 Mar 10;19:35. doi: 10.1186/s12961-020-00651-4 (PMC7948345; doi:10.1186/s12961-020-00651-4)
Supplement: Supplementary file 2 — Additional file 2.The MINDSPACE framework. [file 12961_2020_651_MOESM2_ESM.docx]

**Additional file 2: The MINDSPACE framework**

The MINDSPACE framework was developed by ([Dolan et al., 2010](#_ENREF_88)) as a checklist for policymakers.

| Messenger | we are heavily influenced by who communicates information |
| --- | --- |
| Incentives | our responses to incentives are shaped by predictable mental shortcuts such as strongly avoiding losses |
| Norms | we are strongly influenced by what others do |
| Defaults | we „go with the flow‟ of pre-set options |
| Salience | our attention is drawn to what is novel and seems relevant to us |
| Priming | our acts are often influenced by sub-conscious cues |
| Affect | our emotional associations can powerfully shape our actions |
| Commitments | we seek to be consistent with our public promises, and reciprocate acts |
| Ego | we act in ways that make us feel better about ourselves |
